# Efficacy of lisafentamine treatment in adult patients with comorbid ASD and ADHD: case series

**DOI:** 10.1192/j.eurpsy.2025.1829

**Published:** 2025-08-26

**Authors:** A. Izquierdo De La Puente, S. T. Romero, P. Del Sol Calderon, R. Fernandez Fernandez, E. Antolin Murillo, P. Martinez Marin

**Affiliations:** 1 Psiquiatria; 2 Psicología, Hospital Universitario Puerta de HIerro de Majadahonda; 3 Psiquiatria, Hospital Universitario Infanta Cristina, Madrid, Spain

## Abstract

**Introduction:**

A case series of patients diagnosed with comorbid ASD and ADHD in adulthood is presented.

**Objectives:**

To make a comparison between the situation and social difficulties before and after specific ADHD treatment, in order to reinforce that it is not only a treatment for academic functioning, but also helps social interaction and therefore general functioning.

**Methods:**

Four clinical cases are presented, three females and one male, with a mean age of 45 years. All of them were diagnosed with ASD and comorbid ADHD, with a predominance of impulsive component, in 2023.

All of them, prior to the diagnosis of ASD, were diagnosed with GAD, social phobia or even avoidant personality disorder. For these diagnoses, from a very early age, they received different SSRIs and benzodiazepines with little response.

**Results:**

In the consultation, after taking a complete clinical history, and fundamentally a biographical history, the examination was complemented with ADOS and CPT.

Due to the results obtained in CPT, it was decided to start treatment with lisafentamine between 50 and 70mg DMD.

CPT was performed again, obtaining better results in all cases, with a decrease in impulsivity and attention, mainly. At a subjective level, patients reported a substantial improvement, especially at a social level, which favoured their functioning in basic activities of daily living.

**Conclusions:**

In adults, a correct diagnosis is important given that many of them present symptoms compatible with one or more neurodevelopmental disorders not diagnosed in childhood. Likewise, treatment in accordance with these disorders improves functionality and avoids polypharmacy and side effects of unnecessary treatments.

**Disclosure of Interest:**

None Declared

